# In Vitro Anti-Prostate Cancer Activity of Two Ebselen Analogues

**DOI:** 10.3390/ph13030047

**Published:** 2020-03-17

**Authors:** Katarzyna B. Kaczor-Keller, Anna Pawlik, Jacek Scianowski, Agata Pacuła, Magdalena Obieziurska, Fabio Marcheggiani, Ilenia Cirilli, Luca Tiano, Jedrzej Antosiewicz

**Affiliations:** 1 Department of Bioenergetics and Physiology of Exercise, Medical University of Gdansk, 80-211 Gdansk, Poland; katarzyna.kaczor@gumed.edu.pl; 2 Department of Medical Biology and Genetics, University of Gdansk, 80-308 Gdansk, Poland; anna.pawlik@biol.ug.edu.pl; 3 Department of Organic Chemistry, Faculty of Chemistry, Nicolaus Copernicus University, 87-100 Torun, Poland; jsch@umk.pl (J.S.); pacula@umk.pl (A.P.); magdao@umk.pl (M.O.); 4 Department of Life and Environmental Sciences, Polytechnic University of Marche, 60-100 Anconan, Italy; f.marcheggiani@univpm.it (F.M.); i.cirilli@pm.univpm.it (I.C.); l.tiano@staff.univpm.it (L.T.)

**Keywords:** Organoselenium compound 1, apoptosis 2, ROS 3, DNA damage 4, chemoprevention 5

## Abstract

Scientific research has been underway for decades in order to develop an effective anticancer drug, and it has become crucial to find a novel and effective chemotherapeutics in the case of prostate cancer treatment. Ebselen derivatives have been shown to possess a variety of biological activities, including cytostatic and cytotoxic action against tumor cells. In this study, the cytotoxic effect and anticancer mechanism of action of two organoselenium compounds— (*N*-allyl-1,2-benzisoselenazol-3(2H)-one (N-allyl-BS) and *N*-(3-methylbutyl)-1,2-benzisoselenazol-3(2H)-one) (*N*-(3-mb)-BS)—were investigated on two phenotypically different prostate cancer cell lines DU 145 and PC-3. The influence of analyzed compounds on the viability parameter was also assessed on normal prostate cell line PNT1A. The results showed that both organoselenium compounds (OSCs) efficiently inhibited cancer cell proliferation, whereas normal PNT1A cells were less sensitive to the analazyed ebselen analouges. Both OSCs induced G2/M cell cycle arrest and prompted cell death through apoptosis. The detection of cleaved Poly (ADP-ribose) Polymerase (PARP) confirmed this. In addition, N-allyl-BS and N-(3-m)-b-BS increased the level of reactive oxygen species (ROS) formation, however only N-allyl-BS induced DNA damage. Based on our data, we assume that OSCs’ anticancer action can be associated with oxidative stress induction and inactivation of the Akt- dependent signalling pathway. In conclusion, our data demonstrate that ebselen derivatives showed strong cytotoxic efficiency towards prostate cancer cells and may be elucidated as a novel, potent anticancer agent.

## 1. Introduction

The search for novel and more effective drugs for prostate cancer treatment has been going on for decades, yet the progress in curing tumors is marginal. Recently, prostate cancer has been listed as the second leading cause of male death in the US and western countries, but its incidence is also high in other regions of the world, according to GLOBOCAN statistics [1]. These figures highlight the urge of novel and potent chemotherapeutics against prostate malignancies. 

In particular, over the last decades, much effort has been made to develop new anticancer drugs, which exhibit less toxicity towards noncancerous human cells. The potential therapeutic use of compounds containing selenium and selenium itself has been extensively studied over the years. Selenium-containing compounds, such as ebselen, sodium selenite, diallyl selenite, triphenylselenium chloride, and many others, have been tested. Ebselen [*N*-phenyl-1,2-benzisoselenazol-3(2H)-one] is one of the most extensively studied organoselenium compounds (OSCs). It exhibits versatile biological activities, including anti-inflammatory, anti-bacterial, cytoprotective, neuroprotective, and anticancer activities. Further, this selenium-containing molecule reveals pronounced antioxidant activity, as it mimics glutathione peroxidase, an enzyme that regulates redox homeostasis and protects the cell from oxidative stress. The anticancer activity might play an important role in cancer chemoprevention. 

The anticancer activity of many OSCs is associated with the inhibition of angiogenesis, induction of apoptosis [cleavage of poly(ADP-ribose) polymerase (PARP), activation of caspases, and DNA fragmentation], and cell cycle arrest in human breast, hepatoma, ovarian, prostate, colon, and lung cancer cells, as well as the reduction of oxidative stress [2,3]. Furthermore, methyl selenic acid has been shown to down-regulate the androgen receptor and androgen signaling (via an Akt-dependent pathway) [4]. Currently, it is thought that this mechanism of activity of selenium is closely related to its chemopreventive activity and might be exploited for the development of anticancer therapy [5,6]. It has been also reported that selenium significantly reduces the expression of a prostate-specific antigen by enhancing the recruitment of co-repressors to the promoter of the PSA gene [4]. Collectively, these studies suggest that OSCs show a wide spectrum of anticancer activity, which makes them attractive candidates for exploring their anticancer efficacy.

We have recently synthetized a number of OSCs and demonstrated that some of them show selective anticancer activity [7,8]. In addition, many of these compounds show strong antioxidant properties, which are similar to, or even stronger than, the activity of ebselen. Interestingly, some of these compounds exert a strong cytotoxic effect, despite their antioxidant activity [7,8].

PC-3 and DU145 cells are commonly analyzed cancer cell lines. They are phenotypically different. The PC-3 cell line is negative for a phosphatase and tensin homolog (PTEN) activity. The loss of PTEN function leads to the over-activation of Akt by phosphorylation. Akt is a serine threonine kinase that regulates many processes, including cell cycle progression, apoptosis, cell migration, nutrient metabolism, or angiogenesis [9,10]. The inhibition of Akt signaling leads to apoptosis and growth inhibition, particularly in tumor cells that are characterized by elevated Akt activity. Consequently, Akt is regarded as an attractive candidate for therapeutic intervention. Therefore, PTEN-negative tumors are characterized by a more aggressive phenotype than PTEN-positive tumors and exhibit high metastatic potential [11].

The purpose of the current study was to evaluate the mechanism of anticancer activity of N-allyl-benzisoselenazol-3(2H)-one(N-allyl-BS) and *N*-(3-methylbutyl)-1,2-benzisoselenazol-3(2H)-one (*N*-(3-mb)-BS) in prostate cancer cells, in order to fully assess its potential as an effective antitumor agent. We investigated the effect of the OSCs on the two prostate cancer cell lines, DU145 and PC-3, as well as PNT1A cells, to evaluate its effect on normal cells. We show that both OSCs reduced the survival of cancer cells and induced cell death via apoptosis. This might be an outcome of cell cycle arrest, enhanced reactive oxygen species (ROS) formation, DNA damage, and the inactivation of the Akt signaling pathway.

## 2. Materials and Methods

### 2.1. Synthesis of N-allyl-1,2-benzisoselenazol-3(2H)-one (N-allyl-BS) and N-(3-methylbutyl)-1,2-benzisoselenazol-3(2H)-one (N-(3-mb)-BS)

*N*-allyl-BS was synthetized according to our previously presented protocol [7]. To a solution of allylamine (2.0 mmol) and triethylamine (4.0 mmol) in dichloromethane 2-(chloroseleno)benzoyl chloride (1.0 mmol) was added. The mixture was stirred for 24 h at room temperature, poured on water, and then extracted with DCM. The combined organic layers were dried over anhydrous magnesium sulfate and then evaporated. 1H, 13C, and 77Se NMR spectra confirmed the structure of the obtained pure product (yield 91%). Stock solution of 100 mM *N*-allyl-BS was prepared in 0.1% DMSO and then stored at 4 °C. N-(3-m)-b-BS was synthetized according to our previously presented two-step protocol [12].

### 2.2. Reagents

Culture media, fetal bovine serum, penicillin-streptomycin solution, glutamine, DMSO, and sulforhodamine B (SRB) were purchased from Sigma–Aldrich (St. Louis, MO, USA). Muse kits were obtained from EMD Millipore Bioscience (Billerica, MA, USA). 6-Carboxy-2′,7′-dichlorodihydrofluorescein diacetate (H2DCFDA) was from Molecular Probes (Warsaw, Poland). The antibodies against AKT and phospho-(Ser473)-AKT were from Santa Cruz Biotechnology (Heidelberg, Germany). The PARP antibody was from Cell Signaling Technology (Danvers, MA). Anti-β-actin and anti-rabbit antibodies were from Sigma–Aldrich.

### 2.3. Cell Line and Cell Culture

The DU145 cancer cell line purchased from the American Type Culture Collection (ATCC, Manassas, VA) was maintained in MEME medium that was supplemented with 10% fetal bovine serum, 1% penicillin/streptomycin, 2 mM glutamine, and 1 mM sodium pyruvate. PC-3 cells were also from ATCC. Noncancerous, prostate epithelial PNT1A cells were from the European Collection of Authenticated Cell Cultures (ECACC, France, Paris). The PC-3 and PNT1A cells were cultured in RPMI 1640 medium supplemented with 10% fetal bovine serum and 1% penicillin/streptomycin. All of the cell lines were maintained at 37 °C under 5% CO_2_ in a humidified incubator. In addition, the PC-3 cells were cultured and treated under hypoxic (4% O_2_) condition at 37 °C.

### 2.4. SRB Cell Viability Assay

Cell viability was determined by using the SRB assay. The cells were seeded into 96-well plates (6 ×10^3^ cells/well) in 200 μL of culture medium for 24 h. The cells were then treated with various concentrations (2.5, 5, 10, 20, 30, or 40 μM) of *N*-allyl-BS or *N*-(3-m)-b-BS for additional 24 h. After incubation, the cells were fixed in 20% trichloroacetic acid for 1 h. The plates were then washed with distilled water, air-dried, and stained with 0.4% solution of SRB (Sigma–Aldrich) in 1% acetic acid, for 15 min. The cells were washed four times with 1% acetic acid and then dried. SRB was then solubilized in 10 mM Trisma-base solution and sample absorbance was measured at 570 nm while using an automated microplate reader. The data were obtained from at least three independent experiments with six samples for each treatment.

### 2.5. Western Blot Analysis

The cells were treated with 40 μM *N*-allyl-BS or *N*-(3-mb)-BS for different periods of time and then lysed with a solution of 50 mM Tris–HCl (pH 7.5), 150 mM NaCl, 1% Triton X-100, 0.1% SDS, and protease and phosphatase inhibitor cocktails (Roche Diagnostics). The cell lysates were cleared by centrifugation at 16,000× *g* for 20 min. The proteins were resolved on 10–12% SDS-PAGE and then transferred onto polyvinylidene fluoride membrane, or separated using Trans-Blot^®^ Turbo^TM^ mini-size transfer stacks (Bio-Rad Laboratories, Hercules, California, using Trans-Blot^®^ Turbo^TM^ transfer system M (Bio-Rad Laboratories). The membranes were incubated with a TBST solution containing Tris-buffered saline, 0.05% Tween 20, and 5–10% (w/v) nonfat dry milk, and then exposed to the appropriate primary antibody overnight at 4 °C. After four washes in TBST, the membranes were treated with the appropriate secondary antibody for 1 h at 22 °C. Next, the protein bands were visualized while using ImageQuant LAS 500 (GE Healthcare). The changes in protein levels were quantified by densitometry using the LASImage software. β-Actin was used as a reference control.

### 2.6. Cell Death Assay

The cells were seeded into six-well plates (3 × 10^5^/well) for 24 h. Next, the cells were incubated with 40 μM *N*-allyl-BS or *N*-(3-mb)-BS for 24 h. The medium and trypsynized cells were collected and centrifuged at 300× *g* for 10 min. The early and late apoptotic, and necrotic cells were detected using Muse^®^ cell analyzer, and Muse annexin-V and dead cell assay kit (Muse, Darmstadt, Germany), according to the manufacturer’s instructions.

### 2.7. Cell Cycle Assay

For the experiment, the cells (3 × 10^5^ cells/well) were seeded in six-well plates for 24 h and then treated with 40 μM *N*-allyl-BS or *N*-(3-mb)-BS for 8 h. Subsequently, both floating and attached cells were collected, trypsynized, washed with PBS, and then fixed with 70% ethanol overnight at −20 °C. The cells were stained using the Muse cell cycle kit (Muse), according to the manufacturer’s instructions, and then analyzed by Merck Muse^®^ Cell Analyzer.

### 2.8. Cell Viability Assay

Prior to the experiment, the cells cultured in both normoxic and hypoxic conditions were seeded (3 × 10^5^ cells/well) in six-well plates for 24 h. After 24 h of culture in normal growth medium, the cells were treated with 40 μM *N*-allyl-BS or *N*-(3-mb)-BS for another 24 h. The medium and trypsinized cells were collected and centrifuged at 500× *g* for 5 min. Next, the cells were washed with 1 mL of PBS and then resuspended in 50 μL of fresh medium. In order to determine the viability, 15 μL of the cells were added to freshly prepared ViaCount (merck Millipore) solution (135 μL), according to manufacturer’s instructions, in the final volume of 150 μL for each sample. The cells were analyzed while using Guava Easycyte HT Flow Cytometer.

### 2.9. Detection of Intracellular ROS

The generation of intracellular ROS was determined by flow cytometry with H2DCFDA. Prior to the experiment, cells (3 × 10^5^ cells/well) were seeded in a 6-well plate, allowed to attach overnight, and then exposed to 40 μM *N*-allyl-BS or *N*-(3-mb)-BS for 2 h. Subsequently, the cells were stained with 10 μM H2DCFDA for 30 min. at 37 °C in a complete medium. Floating and attached cells were collected and centrifuged, washed twice with PBS, and then placed on ice. Immediately afterwards, cell fluorescence was analyzed using a BD LSR II flow cytometer. The fluorescence of unstained cells was also measured and subtracted as the background.

### 2.10. Comet Assay

The comet assay was performed to evaluate DNA damage. Briefly, the aliquots of treated and control cells (10,000 cells per sample) were transferred to 1.5-mL centrifugation tubes, and then centrifuged for 10 min. at 800× *g* at 4 °C. The supernatant was removed and the cells were resuspended in 0.7% low-melting agarose of which 0.035 mL was placed on pre-coated, high-throughput comet assay slides (Trevigen). These slides have a clean area separated by silicon barriers to allow simultaneous layering of ten different samples on each slide. The clean areas are manufactured with a dried agarose coating, in order to enhance adhesivity. The microgels on slides were allowed to solidify at 4 °C. Subsequently, the slides were immersed overnight at 4 °C in the dark in ice-cold, freshly prepared lysis solution (2.5 M NaCl, 100 mM Na2EDTA, 10 mM Tris-HCl, 1% Triton X-100, and 10% DMSO, with pH adjusted to 10) to lyse the embedded cells and to allow DNA to unfold. After incubation in the lysis solution, the slides were placed in an alkaline buffer (1 mM Na2EDTA and 300 mM NaOH buffer, pH 7.4 [13] for 30 min. to allow DNA to unwind. Electrophoresis was then performed for 20 min. at 1 V/cm in the same buffer. After neutralization in a Tris buffer (pH 7.5) and dehydration in 75% methanol, DNA on each slide was stained with 0.015 mL of ethidium bromide (20 μg/mL) and then viewed under fluorescent light using an Olympus BX51 fluorescence microscope connected to a computer.

### 2.11. Analysis of the Comet Assay Data

For each sample, 15 randomly acquired images were recorded and processed using custom-made software. The Department of Life and Environmental Sciences developed the software in collaboration with the Engineering Department of the Polytechnic University of Marche [13]. It is based on the Labview programming platform (National Instruments), which enables the automatic identification of comets, thus greatly reducing operator-dependent variability. A key feature of the software is the ability to distinguish the comets from the background, and determine the commonly used DNA damage indices, including tail length, tail intensity, and tail moment. Comet-specific DNA damage indices and images of 150 nucleoids from each slide were fed to a Microsoft Access database. The data and images were then easily traceable for subsequent evaluation and statistical analysis. Three slides were analyzed for each treatment condition and a total of 450 comets were saved.

### 2.12. Statistical Analysis

The data were analyzed using GraphPad Prism 6. T-test, One-way ANOVA, followed by Dunnett’s or Bonferroni’s multiple comparison test were used to determine statistical significance of the differences in measured variables between the control and treated groups. Differences were considered to be significant at P ˂ 0.001, P < 0.01, and P < 0.05.

## 3. Results

### 3.1. N-Allyl-BS and N-(3-mb)-BS Inhibit the Viability of DU145 and PC-3 Prostate Cancer Cell

**Figure 1:**
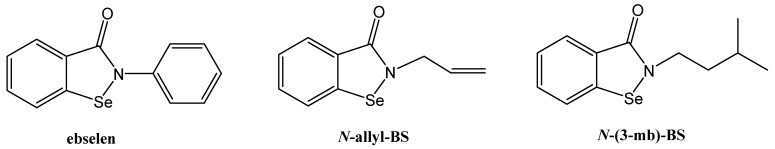
Molecular structure of ebselen, *N*-allyl-1,2-benzisoselenazol-3(2H)-one (*N*-allyl-BS), and *N*-(3-methylbutyl)-1,2-benzisoselenazol-3(2H)-one (*N*-(3-mb)-BS).

**Figure 2:**
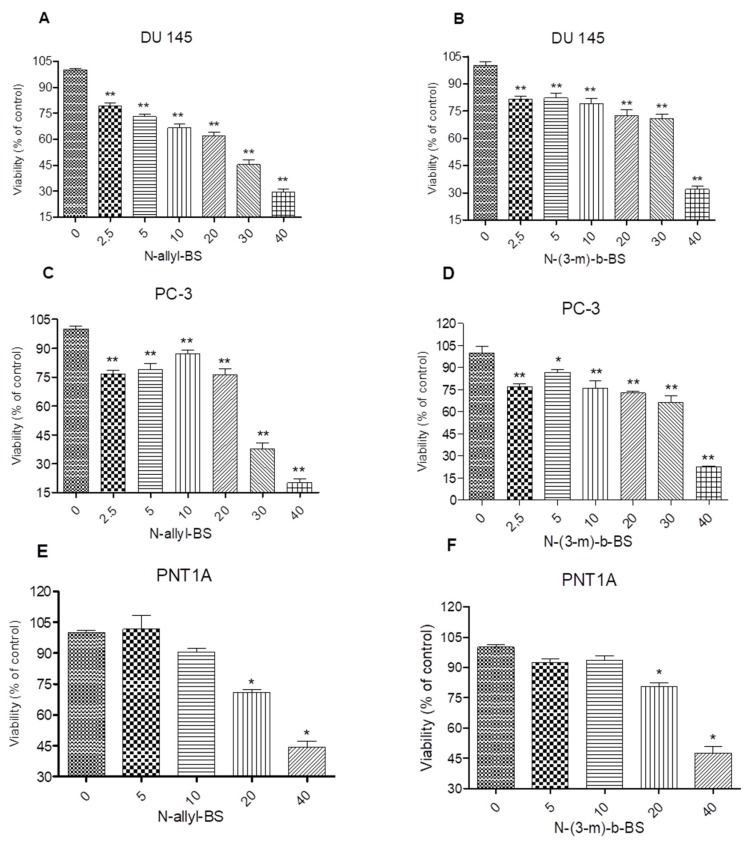
*N*-allyl-BS and *N*-(3-mb)-BS respectively inhibit viability of DU 145 (**A**,**B**) and PC-3 (**C**,**D**) prostate cancer cells in a dose dependent manner, while normal prostate cells PNT1A (**E,F**) are more resistant. The cells were treated with indicated concentrations of *N*-allyl-BS or *N*-(3-mb)-BS for 24 h. Viability was determined using SRB assay. Data are presented as mean ± SE (n = 3); significance of variation was calculated using one-way ANOVA followed by Dunnett’s multiple comparison test. (* P < 0.01) (** P < 0.001).

A series of *N*-alkyl-1,2-benzisoselenazol-3(2H)-ones were synthesized and their potential anticancer activity tested. Two compounds with the strongest anti-proliferative activity against prostate cancer cells were selected and further examined (Figure 1) [7]. The anticancer capacity of the *N*-allyl-BS and *N*-(3-mb)-BS was studied while using two phenotypically different cancer cell lines: PTEN-positive (DU145) with normal Akt kinase protein levels and PTEN-negative (PC-3) with high Akt kinase activity. The cells were exposed for 24 h to increasing concentrations (2.5–40 μM) of the analyzed compounds and the cytotoxic effect of *N*-allyl-BS and *N*-(3-mb)-BS was evaluated using the SRB assay. The inhibition of cell proliferation was significant at all tested concentrations in both cancer cell lines. The highest anti-proliferative activity was observed after incubation in the presence of 40 μM, with the viability of DU145 and PC-3 cells reduced by 60 and 80%, respectively (Figure 2A–D). Further, the two-way Anova analysis indicates that the cytotoxicity of *N*-allyl-BS and *N*-(3-mb)-BS in the normal prostate cell line (PNT1A) was significantly lower when compared with the cancer cell lines, especially at 40 μM (p < 0.05) (not shown).

### 3.2. N-allyl-BS and N-(3-mb)-BS Induce G2/M Cell Cycle Arrest in Prostate Cancer Cells

**Figure 3:**
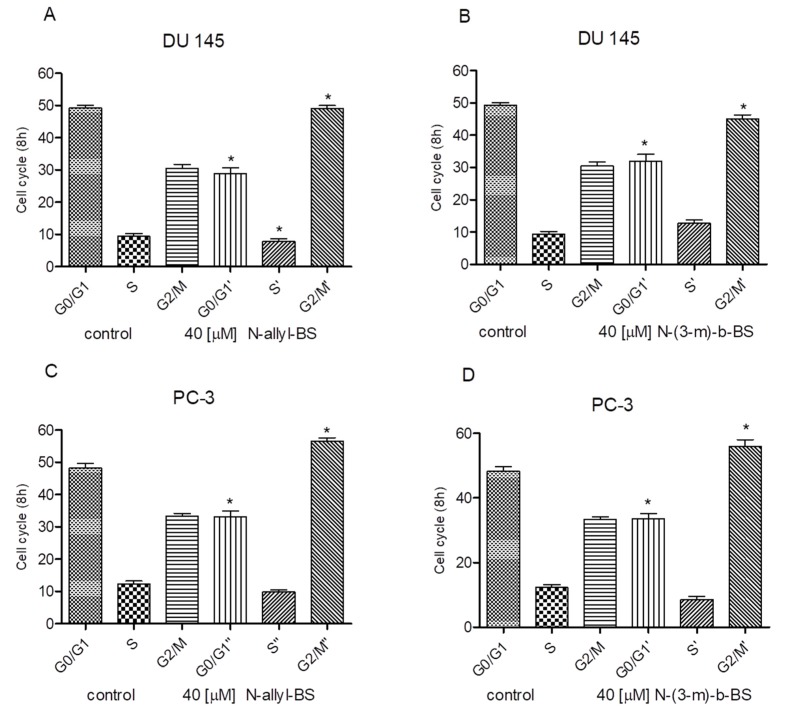
40 µM *N*-allyl-BS and *N*-(3-mb)-BS respectively induce G2/M cell cycle arrest in prostate cancer cells DU145 (**A**,**B**), PC-3 (**C**,**D**), after 8h treatment. The data are presented as mean ± SE (n = 3). Significance of variations compared with control was calculated using one-way ANOVA followed by Bonferroni’s multiple comparison test. (*P < 0.01).

Next, the inhibitory effect of *N*-allyl-BS on the cell cycle was determined while using flow cytometry. The experiment revealed that the 8h incubation of tumor cells with 40 μM *N*-allyl-BS and *N*-(3-mb)-BS significantly increased the percentage of cells in the G2/M phase. This phenomenon was observed in both cancer cell lines tested (Figure 3A–D). Therefore, *N*-allyl-BS and *N*-(3-mb)-BS induced G2/M cell cycle arrest. Moreover, the cytostatic effect on PC-3 cells was stronger than that exerted on DU145 cells (Figure 3C,D).

### 3.3. N-allyl-BS and N-(3-mb)-BS Induce Apoptosis and Necrosis of Cancer Cells

**Figure 4:**
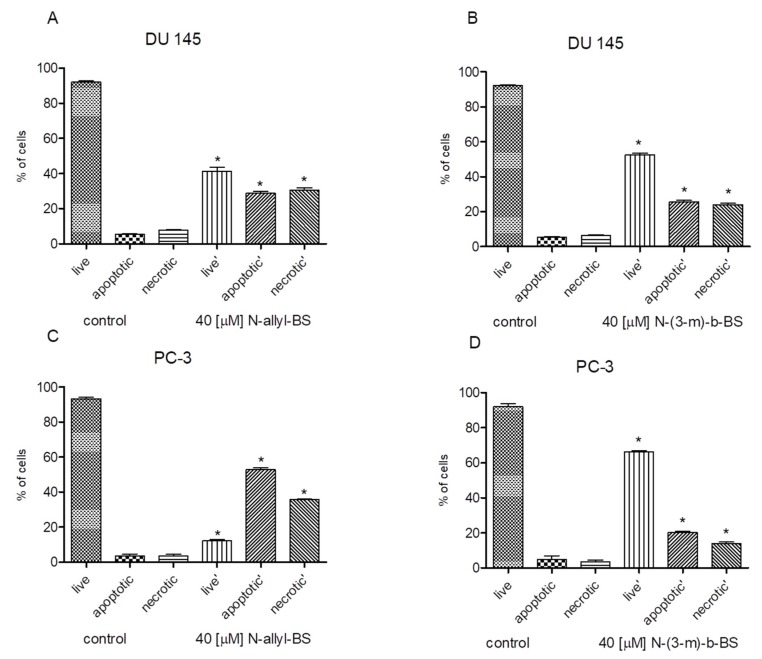
40 µM *N*-allyl-BS (**A,C**) and *N*-(3-mb)-BS (**B,D**) respectively induce apoptosis and necrosis of cancer cells after 24 h treatment. Percentage of live, apoptotic and necrotic cells was determined by flow cytometry. The results are presented as a mean ± SE of three independent experiments. Significance of variations was determined by one-way ANOVA followed by Bonferroni’s multiple comparison test. (* P < 0.01).

**Figure 5:**
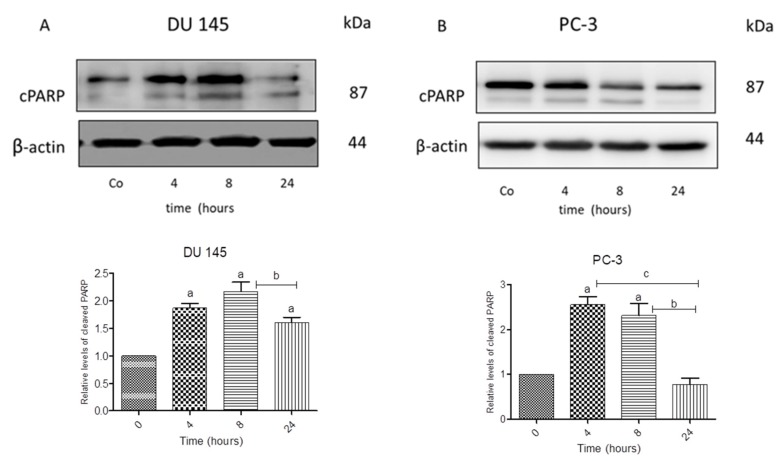
Western blot analysis of cleaved PARP in DU 145 (**A**) and PC-3 cells (**B**) treated with 40 µM *N*-allyl-BS for indicated time points. β-actin was used as a lane loading control. The results are presented as mean ± SE of three independent experiments. The statistical significance of differences between respective samples was determined by one-way ANOVA followed by Bonferroni’s multiple comparison test, where (**A**) indicates significant difference between control and treated cells P < 0.05 and (b, c) indicates significant difference between 4, 8, and 24 h of 40 µM *N*-allyl-BS-treated cells P < 0.05.

**Figure 6:**
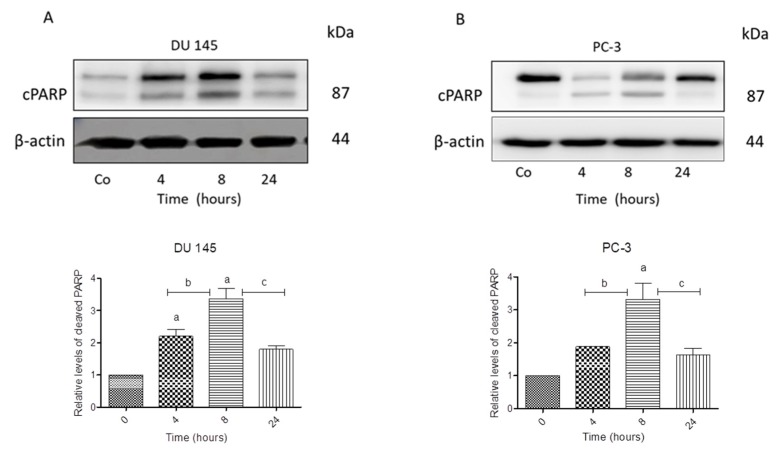
Western blot analysis of cleaved PARP in DU 145 (**A**) and PC-3 cells (**B**) treated with 40 µM and *N*-(3-mb)-BS, respectively, for indicated time points. β-actin was used as a lane loading control. The results are presented as mean ± SE of three independent experiments. The statistical significance of differences between respective samples was determined by one-way ANOVA followed by Bonferroni’s multiple comparison test, where (a) indicates significant difference between control and treated *N*-(3-mb)-BS treated cells P < 0.05.

We next investigated whether *N*-allyl-BS and *N*-(3-mb)-BS could induce cell death in prostate cancer cells since the DU145 and PC-3 cells differ with respect to the Akt kinase activity. Both cancer cell lines were treated for 24 h with 40 μM *N*-allyl-BS or *N*-(3-mb)-BS and then analyzed using flow cytometry. Indeed, *N*-allyl-BS induced apoptosis and necrosis in DU145 and PC-3 cells (Figure 4A–D). The pro-apoptotic effect of *N*-allyl-BS was more pronounced in PC-3 cells than in DU145 cells (Figure 4A,C The level of caspase-dependent cleaved PARP, an apoptosis marker and enzyme responsible for DNA repair, was evaluated in cells exposed to *N*-allyl-BS or *N*-(3-mb)-BS for different periods of time to confirm that *N*-allyl-BS and *N*-(3-mb)-BS indeed caused cell death via apoptosis. The highest level of cleaved PARP was observed after 4 h of treatment with 40 μM *N*-allyl-BS or *N*-(3-mb)-BS in PC-3 cells, and after 8 h of treatment in DU145 cells, as shown in (Figure 5A,B and Figure 6A,B). Interestingly, the level of PARP cleavage significantly dropped after 24 h treatment.

### 3.4. N-Allyl-BS and N-(3-mb)-BS Respectively Enhance ROS Generation in Cancer Cells

**Figure 7:**
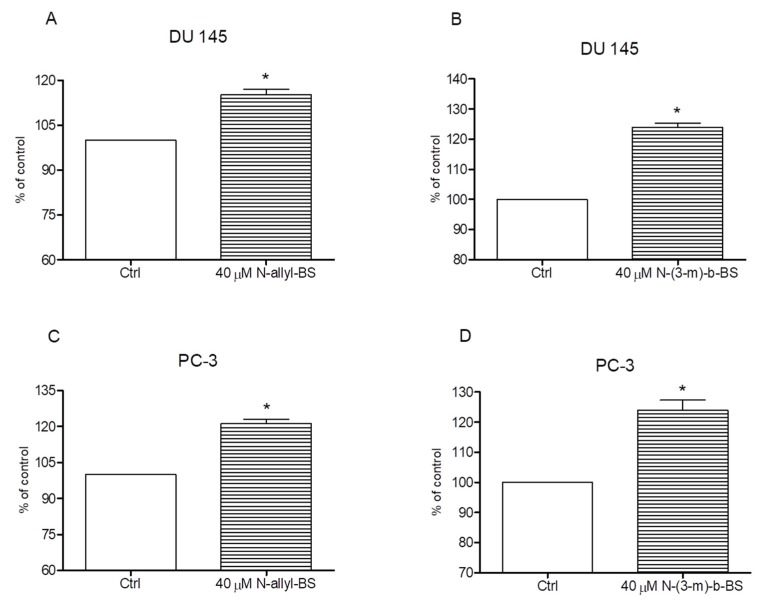
40 µM *N*-allyl-BS and *N*-(3-mb)-BS, respectively, enhance reactive oxygen species (ROS) generation in DU 145 (**A**,**B**) and PC-3 (**C**,**D**) cancer cells after 2 h of treatment. The amount of ROS generation was assessed using flow cytometry. Results are presented as a mean ± SE of three independent experiments. The significance of differences compared to control was calculated using student T test. (*P < 0.01).

The intracellular level of ROS was determined to investigate whether oxidative stress was involved in the cytotoxic activity of *N*-allyl-BS and *N*-(3-mb)-BS. A significant increase in ROS generation was apparent after 2h treatment with 40 μM *N*-allyl-BS or *N*-(3-mb)-BS in the two cancer cell lines tested (Figure 7A–D). At that time point, *N*-allyl-BS and *N*-(3-mb)-BS induced a 20% increase in ROS production in DU145 and PC-3 cells.

### 3.5. N-allyl-BS Induces DNA Damage

**Figure 8:**
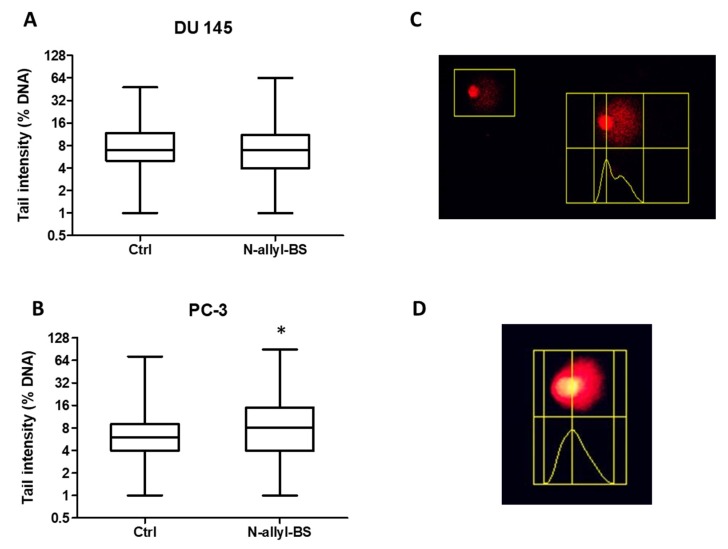
The influence of 40 µM *N*-allyl-BS on DNA damage in DU 145 (**A**) and PC-3 (**B**) cells after 24 h treatment was determined using comet assay. The results are presented asthe percentage of DNA in Tail Intensity ± SE (n = 3). Significance of differences compared with control was calculated by student T test. (* P < 0.05). Representative pictures of DU 145 (**C**) and PC-3 (**D**) cells in the upper 90th percentile of tail intensity.

The comet assay was performed on DU145 and PC-3 cell lines to elucidate whether the increased cell death and oxidative damage via elevated ROS generation was linked with DNA damage. The cells that were treated for 24 h with 40 µM *N*-allyl-BS concentration efficiently induced DNA damage in PC-3 cells, as highlighted by a significant increase of the upper third (+33%) and fourth quartile (+88%) of tail intensity, a parameter indicating the percentage of DNA fluorescence in the tail of comet images proportional to the amount of damaged DNA, on the contrary in the same experimental conditions N-allyl-BS had no significant effects on the DU145 cells (Figure 8A,B). In addition, *N*-allyl-BS induced DNA damage already after 8 h of treatment in PC-3 cells (not shown). *N*-(3-mb)-BS did not induce DNA damage in PC-3 or in DU 145 cells (not shown).

### 3.6. The Cytotoxic Activity of N-Allyl-BS and N-(3-mb)-BS are Similar under Hypoxic and Normoxic Conditions

**Figure 9:**
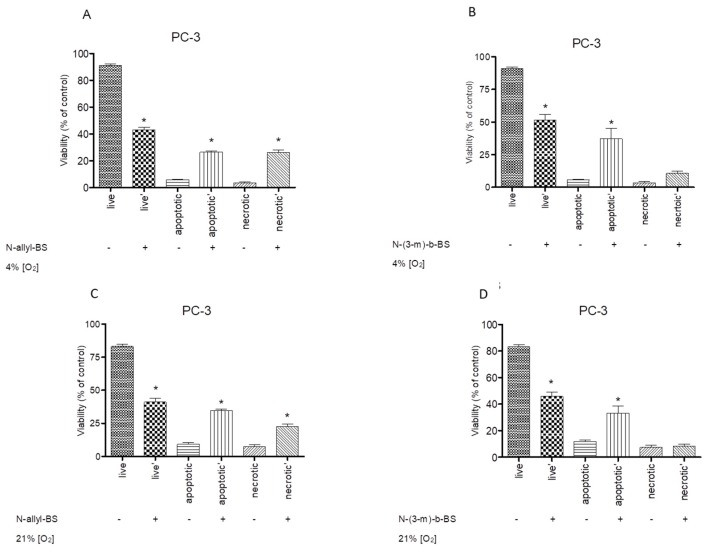
40 µM *N*-allyl-BS and *N*-(3-mb)-BS exhibit similar cytotoxic activity towards PC-3 cells both in hypoxic and normoxic conditions (**A**–**D**). The cells were treated with indicated concentration of *N*-allyl-BS or *N*-(3-mb)-BS for 24 h. The viability was assessed using flow cytometry. Data are presented as mean ± SE (n = 3); significant difference was calculated using one-way ANOVA followed by Dunnett’s multiple comparison test (* P < 0.01).

Cells growing within a tumor are exposed to a much lower concentration of oxygen than cells growing in cell cultures (REF). Cytotoxicity experiments were performed in an atmosphere of 4 and 21% oxygen concentration to test whether different oxygen concentrations affected cell sensitivity to *N*-allyl-BS. The cells were treated for 24 h with 40 μM *N*-allyl-BS and *N*-(3-mb)-BS under hypoxic and normoxic conditions. A similar fraction of apoptotic and necrotic cells was observed under the two conditions (Figure 9A–D). Hence, a low oxygen concentration did not affect the cytotoxic activity of *N*-allyl-BS.

### 3.7. N-Allyl-BS and N-(3-mb)-BS Reduce Akt Phosphorylation

**Figure 10:**
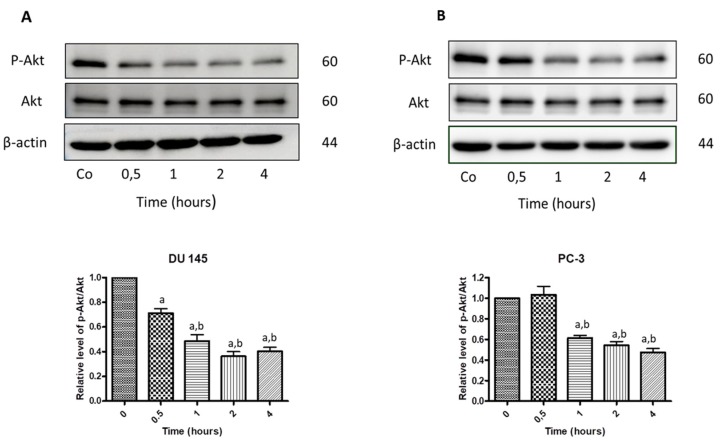
Western blot analysis of p-Akt and Akt kinase in DU145 (**A**) and PC-3 (**B**) cells treated treated with 40 µM *N*-allyl-BS for indicated time points. β-actin was used as a lane loading control. Results are presented as mean ± SE (n = 3). The statistical significance of differences between respective samples was determined by one-way ANOVA followed by Bonferroni’s multiple comparison test, where (a) indicates significant difference between control and treated cells P < 0.01 and (b, c) indicates significant difference between 4, 8, and 24 h of 40 µM *N*-allyl-BS-treated cells P < 0.01.

**Figure 11:**
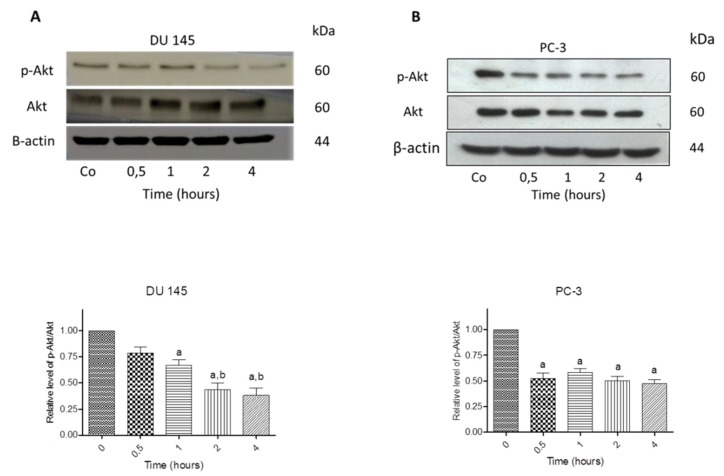
Western blot analysis of p-Akt and Akt kinase in DU145 (**A**) and PC-3 (**B**) cells treated treated with 40 µM *N*-(3-mb)-BS for indicated time points. β-actin was used as a lane loading control. Results are presented as mean ± SE (n = 3). The statistical significance of differences between respective samples was determined by one-way ANOVA followed by Bonferroni’s multiple comparison test, where (a) indicates significant difference between control and treated cells P < 0.01 and (b, c) indicates significant difference between 4, 8, and 24 h of 40 µM *N*-(3-mb)-BS -treated cells P < 0.01.

Decreased Akt phosphorylation induces cell death in prostate cancer cells [14]. The cells were treated with 40 μM *N*-allyl-BS (Figure 10) or *N*-(3-mb)-BS (Figure 11) for different time periods and Akt phosphorylation was analyzed by western blotting to examine whether *N*-allyl-BS and *N*-(3-mb)-BS decreased the level of phosphorylated Akt (p-Akt). Indeed, Akt phosphorylation was slightly reduced after 1-h treatment and highly reduced after 4-h treatment, in the case of N-allyl-BS (Figure 10a,b), however *N*-(3-mb)-BS already slightly reduced the level of Akt phosphorylation after 30 min. of treatment (Figure 11a,b). The changes in Akt phosphorylation were similar in the DU145 and PC-3 cells.

## 4. Discussion

In the current study, we investigated the cytotoxic and antitumor potential of *N*-allyl-BS and *N*-(3-mb)-BS against two phenotypically different cancer cell lines, the PTEN-positive DU145 cells and PTEN-negative PC-3 cells, and against a healthy epithelial cell line PNT1A. We have previously shown that *N*-allyl-BS, which is one of recently synthesized OSCs, exerts a strong antiproliferative effect on prostate and breast cancer cells [7,8]. In the present study, we confirmed these observations by studying the cytotoxic effect of *N*-allyl-BS in prostate cancer cells. Here, we demonstrated that both tested tumor cell lines were sensitive to the analyzed compounds. In addition, the DU 145 and PC-3 cells were more sensitive to *N*-allyl-BS and N-(3-m)-b-BS than the non-tumor cell line PNT1A. As mentioned above, the PC-3 cells are PTEN-negative and it has been demonstrated that the growth of cancer cells depends mainly on the activation of the Akt pathway [15]. Hence, we hypothesized that some of the *N*-allyl-BS and *N*-(3-mb)-BS anticancer activities (apoptosis induction and/or cell-cycle arrest) could be associated with the inhibition of Akt kinase. In fact, the presented data confirmed that the levels of the active form of Akt (p-Akt) were significantly reduced in the DU145 and PC-3 cells treated with both compounds. This suggested that the higher sensitivity of PC-3 than DU145 cells to *N*-allyl-BS was associated with Akt inactivation. Furthermore, PC-3 cells accumulated significant DNA damage upon *N*-allyl-BS exposure, as determined by the comet assay, while the changes in DU145 cells did not reach statistical significance. Interestingly, *N*-(3-mb)-BS did not induce DNA damage in both cancer cell lines, which indicates the different molecular mechanism of cell death. As above, this suggested that *N*-allyl-BS induced genotoxic stress in PC-3 cells. As ROS can potentiate DNA damage, the effect of the studied compounds on ROS production has been evaluated. The data clearly shows that both OSCSs induce ROS formation, in both cancer cell lines. Therefore, the differences in the extent of DNA damage could not be explained by changes in ROS production. Interestingly, the hyperactivity of Akt might also affect DNA repair. It has been demonstrated that PTEN-deficient HCT116 cells that exhibit high Akt activity fail to repair DNA as efficiently as the wild type after irradiation [16]. In PTEN-deficient cells, the DNA damage response complex MRE11 is highly unstable, and this might explain impaired DNA repair in these cells [16]. Consequently, it can be concluded that *N*-allyl-BS induced genotoxic stress; however, that was only apparent in PC-3 cells, in which DNA damage repair could be impaired. However, this was not confirmed with PC-3 cells that were treated with *N*-(3-mb)-BS, since no significant DNA damage was observed. Most of the data obtained on cell culture, including our own, are performed in 21% oxygen, while the tissue oxygen level is between 3 to 8% and there are some evidences that the high oxygen concentration routinely used in cell culture might lead to altered cellular pathways in vitro [17,18]. Hence, another goal of the current study was to compare the cytotoxicity of *N*-allyl-BS and *N*-(3-mb)-BS towards prostate cells that were cultured under 21% and 4% oxygen. We hypothesized that the cytotoxic activity of both organoselenium compounds would be lower under 21% oxygen (because of adaptation to greater free radical formation under such conditions) than under 4% oxygen [19,20]. Nevertheless, this assumption was not confirmed, as the cytotoxicity of N-allyl-BS and *N*-(3-mb)-BS was the same under 21% oxygen and 4% oxygen. Previously, we demonstrated that, in vitro, N-allyl-BS exhibits higher antioxidant activity than ebselen in vitro [7]. Conversely, in the current study, we observed that N-allyl-BS induced cell death via apoptosis and enhanced ROS formation. However, the cellular sources and mechanisms underpinning the enhanced ROS generation are unknown. Santofimia-Castano and co-workers [21] reported that ebselen induced apoptosis in pancreatic AR42J cancer cells by increasing mitochondrial ROS production. These data are in agreement with previous studies that showed that ebselen and selenadiazole, which are well-defined antioxidants, induce ROS production in cancer cells [22,23]. The use of apoptosis- and necrosis-inducing agents is being increasingly as a therapeutic approach in cancer treatment, because the induction of these processes would result in a pronounced reduction of tumor mass. In the current study, we showed that *N*-allyl-BS and *N*-(3-mb)-BS induce apoptotic and necrotic cell death, which was confirmed by flow cytometry and western blot analysis of cleaved PARP levels. PARP is an abundant enzyme that is present in all somatic cells. It detects and signals DNA damage to the cellular repair mechanisms. It is well established that PARP cleavage, as catalyzed by caspase 3, occurs during programmed cell death that is induced by a variety of apoptotic stimuli [24]. In the current study, western blot analysis of an *N*-allyl-BS and *N*-(3-mb)-BS—induced PARP cleavage in prostate cancer cells revealed the extensive degradation of PARP after 4-h and 8-h exposure of both cancer cell lines to this compound. These results are in agreement with data showing that ebselen promoted apoptosis in cancer cells by an intrinsic pathway [22]. However, the level of cleaved PARP dramatically decreased after 24-h exposure of PC-3 and DU145 cells to *N*-allyl-BS and *N*-(3-mb)-BS. This phenomenon might be a consequence of necrosis induction. It has been proven that depletion of intracellular ATP levels switches the energy-requiring apoptotic cell death to necrosis [25]. It has been also reported that, during necrosis, further PARP cleavage occurs, with small PARP fragments (Mr 50,000, 40,000, and 35,000) being detected [26]. Although, necrosis, as an uncontrolled modality of cell death, is generally associated with damage to peripheral tissues and increased systemic inflammation; recent observations highlight a positive role of necrosis induction during cancer therapy. In fact, HMGB1 (biochemical marker of necrosis) might be capable of initiating antitumor immunity. Moreover, in vivo studies suggest that immunization with HMGB1 can enhance antitumor immunity against poorly immunogenic apoptotic tumors [27].

## 5. Conclusions

In conclusion, the presented data demonstrate that *N*-allyl-BS and *N*-(3-mb)-BS are active cytotoxic compounds that can inhibit the growth of prostate cancer cells. The cytotoxicity mechanism is different in DU145 and PC-3 cells. Both of the compounds induce oxidative stress, however DNA damage was only observed in PC-3 after treatment with N-allyl-BS. Interestingly, partial oxygen pressure had no effect on the cytotoxic activity of the studied compounds.
